# Lateral Flow Immunoassay for the Detection of Panton-Valentine Leukocidin in *Staphylococcus aureus* From Skin and Soft Tissue Infections in the United Arab Emirates

**DOI:** 10.3389/fcimb.2021.754523

**Published:** 2021-10-18

**Authors:** Abiola Senok, Stefan Monecke, Rania Nassar, Handan Celiloglu, Sreeraj Thyagarajan, Elke Müller, Ralf Ehricht

**Affiliations:** ^1^ College of Medicine, Mohammed Bin Rashid University of Medicine and Health Sciences, Dubai, United Arab Emirates; ^2^ Department of Optical Molecular Diagnostics and System Technology, Leibniz Institute of Photonic Technology (IPHT), Jena, Germany; ^3^ InfectoGnostics Research Campus, Jena, Germany; ^4^ Institute for Medical Microbiology and Virology, Dresden University Hospital, Dresden, Germany; ^5^ Oral and Biomedical Sciences, School of Dentistry, Cardiff University, Cardiff, United Kingdom; ^6^ Department of Pathology & Laboratory Medicine, Mediclinic City Hospital, Dubai, United Arab Emirates; ^7^ Institute of Physical Chemistry, Friedrich-Schiller University, Jena, Germany

**Keywords:** lateral flow immunoassay, *Staphylococcus aureus*, Panton Valentine leucocidin, skin and soft tissue infection, DNA microarray

## Abstract

**Introduction:**

Panton Valentine leukocidin (PVL) is a virulence factor which is associated with methicillin sensitive and resistant *Staphylococcus aureus* (MSSA/MRSA) causing skin and soft tissue infections (SSTI). This study aimed to evaluate a novel lateral flow immunoassay (LFI) for PVL detection in *S. aureus* cultures and to describe their genotypic characterization.

**Methods:**

The study was carried out from January-August 2020 in Dubai, United Arab Emirates. *S. aureus* isolates associated with SSTI were tested for PVL detection using LFI. DNA microarray-based assays were used for molecular characterization including detection of *pvl* genes.

**Results:**

One-hundred thirty-five patients with a clinical diagnosis of SSTIs were recruited. Sixty-six patients received antibiotics, mostly beta lactams (n=36) and topical fusidic acid (n=15). One-hundred twenty-nine isolates (MRSA: n=43; MSSA: n=86) were tested by LFI and DNA microarrays. All 76 (58.9%) isolates which were unambiguously negative for the PVL in LFI were negative for *pvl* genes using the DNA microarray. All the LFI PVL positive isolates (n=53) had *pvl* genes detected. This translates into 100% each for sensitivity, specificity, positive and negative predictive values for the LFI. The LFI typically takes about 15 min inclusive of a 10 min incubation period. Predominant *S. aureus* clonal complexes (CC) were CC30 (n=18), CC22 (n=13), CC5 (n=12), CC1 (n=11), CC152 (n=8), CC15 (n=7); CC97 (n=7); CC8 and CC20 (n=6 each). Among MRSA, the proportion of pvl-positives (35/43; 81%) was higher than among MSSA (n/N=18/86; 21%). The fusidic acid resistance gene *fusC* was detected in 14 MRSA (33%) compared to 8 MSSA (9%). A co-carriage of *fusC* and *pvl* genes was present in 7 MRSA and in one MSSA.

**Conclusion:**

LFI shows excellent diagnostic accuracy indices for rapid identification of PVL in MSSA/MRSA in a setting with high prevalence of *pvl* ^+ve^ strains. The high occurrence of *pvl* and *fusC* genes in MRSA strains causing SSTI is of concern and needs constant surveillance.

## 1 Introduction

Methicillin sensitive and resistant *Staphylococcus aureus* (MSSA/MRSA) are aetiological agents of chronic/recurrent skin and soft tissue infections (SSTI). The spectrum of infections which may be community or hospital acquired range from impetigo and folliculitis to more serious surgical site infections. Global data indicates an increasing trend of incidence and severity of SSTIs caused by MRSA isolates (Olaniyi et al., 2017). Panton Valentine leukocidin (PVL) is a virulence factor which is associated with *S. aureus* strains causing SSTI and severe forms of community acquired pneumonia (Gosbell, 2005; Wannet et al., 2005; Yamasaki et al., 2005; Marazza et al., 2007; Masiuk et al., 2010; Gillet et al., 2021). PVL consists of two distinct components which form polymeric pores in the membranes of white blood cells which leads to cell death. This might explain the higher risk of complicated SSTI infection associated with PVL positive *S. aureus* strains (Szmigielski et al., 1998; Kaneko and Kamio, 2004). The *pvl* genes, *lukS-PV* and *lukF-PV*, are phage borne (Narita et al., 2001; Boakes et al., 2011), and since they are mobile, they can be found in different, unrelated lineages of *S. aureus* (Monecke et al., 2007; Monecke et al., 2011a).

Detection of *pvl* genes in *S. aureus* is usually done by molecular methods and is not routinely offered by most diagnostic laboratories. Findings from a meta-analysis report suggests that PVL-producing *S. aureus* are associated with SSTIs with increased risk for surgical intervention (Shallcross et al., 2013). In light of this, a rapid and affordable method for the identification of PVL in *S. aureus* strains isolated from SSTI, which can be carried out without the need for expensive molecular assays is desirable for the diagnostic microbiology laboratory.

This is particularly pertinent in settings such as the Arabian Gulf region with high prevalence of *pvl* ^+ve^ *S. aureus* (Senok et al., 2016b; Senok et al., 2019b; Senok et al., 2020a). The countries of the Arabian Gulf region are a global hub for travel and commerce with tourists, pilgrims, and expatriate workers from all across the world. This includes regions from which nothing is known on MSSA/MRSA prevalence and *S. aureus* population structure. Indeed, with the cosmopolitan character of the United Arab Emirates (UAE) and reported high biodiversity of MSSA and MRSA including high prevalence of strains with *pvl* genes, it represents a suitable study setting with regard to *S. aureus* toxins and typing (Senok et al., 2020a; Senok et al., 2020b).

In this study, we describe the application of a novel lateral flow immunoassay for rapid PVL detection in *S. aureus* directly from bacterial cultures and in addition, we present data on the molecular characterization of a collection of MSSA/MRSA strains associated with SSTI.

## 2 Methods

### 2.1 Study Site

The study was carried out from January-August 2020 at Mediclinic hospitals and clinics in Dubai, United Arab Emirates. *S. aureus* isolates studied were those associated with SSTI and identified as part of routine diagnostics in the microbiology laboratory during the study period. Only isolates from patients with diagnosis of furunculosis or carbuncles, cutaneous abscesses; or other conspicuous or severe skin and soft tissue infections such as mastitis or necrotising fasciitis; chronically purulent and painful “spider bites”; recurrent or chronic skin- and soft tissue infections were eligible for inclusion. Demographic and clinical data including age, gender, history of recurrence, animal contact, insect bite, travel history and antibiotic use were obtained.

### 2.2 Bacterial Isolates

Only one isolate per patient was included. In a single case, two genotypically different isolates from one sample were considered. *S. aureus* isolates obtained from screening samples (nasal, buccal, axillary, rectal swabs), from diabetic foot ulcers and for other clinical indications were excluded. Identification of *S. aureus*, confirmation of methicillin resistance and antibiotic susceptibility testing were carried using the VITEK 2 automated platform (bioMérieux, Marcy-l’Étoile, France) in accordance to manufacturer’s instructions and Clinical and Laboratory Standards Institute guidelines (CLSI, 2017). Isolates meeting the inclusion criteria were studied for lateral flow immunoassay and molecular characterization. Ethical approval was obtained from Mohammed Bin Rashid University, and Mediclinic Hospitals ethics committees (MBRU-IRB-2020-003 and MCME.CR.98.MCIT.2020).

### 2.3 Lateral Flow Immunoassay

#### 2.3.1 Principle of the Test 

**Figure 1:**
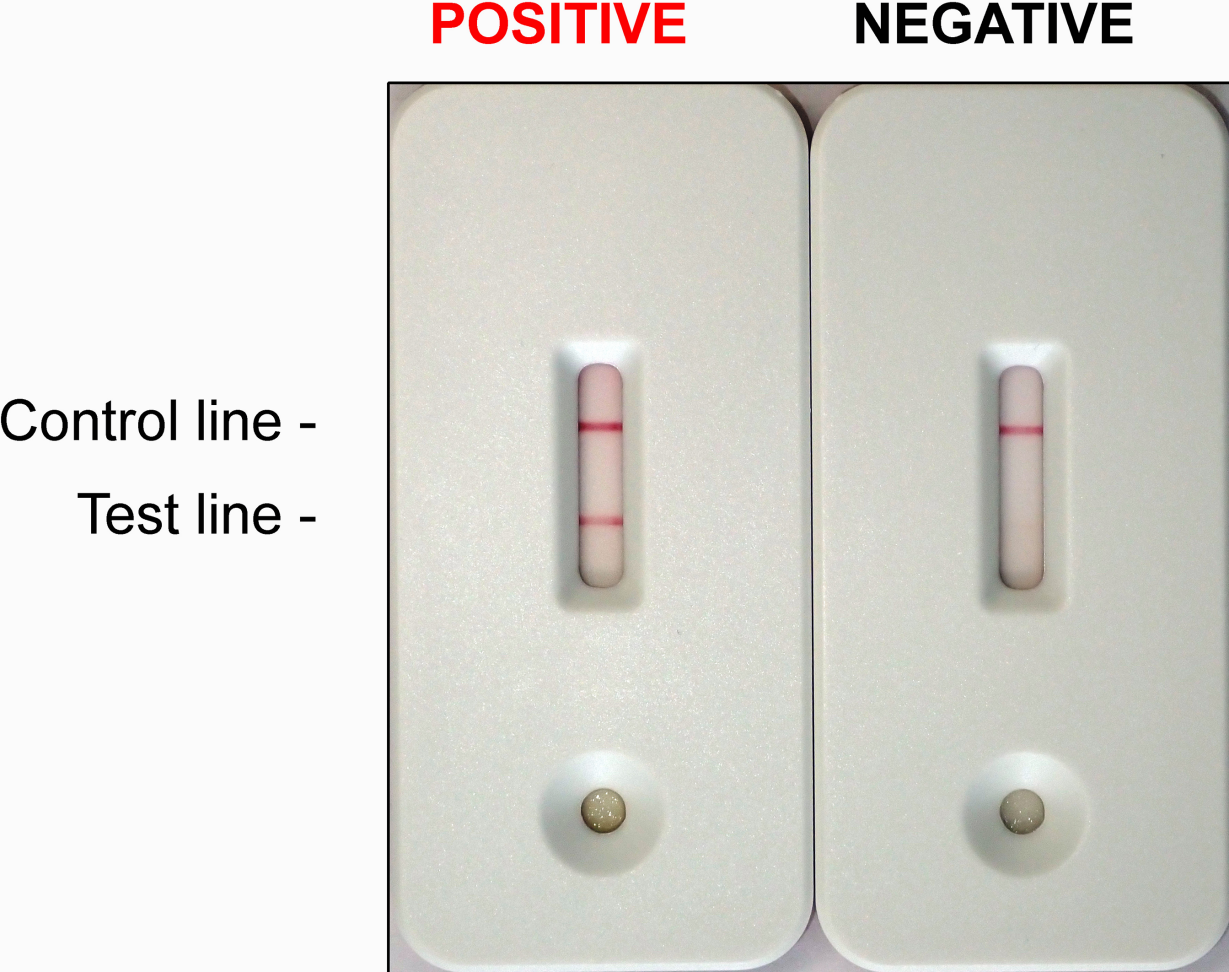
*Staphylococcus aureus* Panton Valentine leukocidin Lateral Flow Immunoassay. A positive and a negative test. Lines must appear within 10 min of incubation at room temperature. Red lines appearing in the TEST line region (TEST) and in the CONTROL line region (CONTROL) indicate together a POSITIVE result (LEFT). The TEST line can be weaker than the CONTROL line. A red line appearing in the CONTROL line region (CONTROL) serves as the internal control. It confirms sufficient sample volume and correct procedural technique. If the CONTROL line was absent, the test is to be regarded as INVALID and should be repeated. A clear background is also required. If only the CONTROL line appears (RIGHT), the test is NEGATIVE.

The core of the lateral flow immunoassay consists of a porous nitrocellulose membrane on which PVL-specific antibodies are immobilized. The antibodies were generated using conventional method using mouse hybridoma cells, and the immunization was performed with recombinant lukF-PV that was produced as previously described (Köhler and Milstein, 2005; Stieber et al., 2014). In addition to the nitrocellulose membrane, other differently functional, successively overlapping membranes are fixed on a self-adhesive plastic card (**Figure 1**). These membranes enable the migration of the soluble proteins from the culture material through the porous structures. Before the sample migrates through the nitrocellulose membrane, it is applied to a sample pad, which ensures even distribution and pre-treatment of the sample. The sample is transported from the sample pad to the adjoining conjugate pad, into which PVL-specific antibodies labeled with dyed particles have been introduced. These conjugates bind specifically to PVL in the sample and are required for visualization on the test line. From there, the complexes comprising dyed particles, antibodies and PVL molecules are led into the nitrocellulose membrane, on which a colored band is generated by analyte enrichment due to the immobilized PVL-specific antibodies, which capture the complexes. Not bounded dyed particle conjugates bind to a second line on the nitrocellulose membrane and form a second reaction-independent control that indicates the functionality of the test. Finally, the sample is passed on to the wicking pad located behind the control zone and picked up there to prevent backflow. The appearance of a visual test band indicates a positive result, and the absence of the test band indicates a negative result. The test is considered invalid if the control band does not appear (**Figure 1**).

#### 2.3.2 Lateral Flow Immunoassay Tests

*S. aureus* isolates meeting the inclusion criteria were sub-cultured on Columbia Blood Agar, incubated overnight or up to 24 hrs at 37°C ± 2°C. Fresh (overnight to 24 hours) cultures were used for the test. One inoculation loop (≥ ~10 µl) full of culture material was inoculated into 300 µl of the kit buffer and vortexed for 15–30 sec until cell suspension was homogenous. The buffer with the suspended inoculum was centrifuged at 2,000 g for 30 sec, then 100 µl of the supernatant was pipetted onto the sample well of the test device, incubated for 10 min at room temperature. The appearance of the visual test band was noted and the image of the result on the lateral flow device was photographed. The appearance of a visual test band indicates a positive result, its absence is indicative of a negative result and the test is considered invalid if the control band does not appear. The image of the test result was independently reviewed by two researchers (S.M & R.E) who were blinded to the DNA microarray result.

### 2.4 DNA Microarray

Molecular characterization was carried out using the StaphyType DNA microarray (Abbott [Alere Technologies GmbH], Jena, Germany) and the INTER-ARRAY Genotyping Kit *S.aureus* (Inter-Array GmbH, Bad Langensalza, Germany) for the detection of species markers as well as of virulence and resistance genes. The previously described probes, primers, and procedures were used (Monecke et al., 2011a; Monecke et al., 2011b; Monecke et al., 2016). SCC*mec* subtyping using a second array was carried out on selected isolates as previously described (Monecke et al., 2016). Microarray images were taken, and analysis was carried out using the dedicated reader and software (Alere Technologies) as previously described (Monecke et al., 2011a; Monecke et al., 2011b; Monecke et al., 2016). The microarray harbored probes for both, *lukS-PV and lukF-PV* thus facilitating molecular confirmation of the lateral flow immunoassay.

## 3 Results

A total of 135 patients were recruited with mean age ( ± SD) 27.7 ( ± 17.9) years and majority (n=71) were females. Most of the patients (n=130) were from the outpatient setting and 20.7% (n/N=28/135) gave a history of recurrent skin infections. None of the patients reported similar infections in family members. Clinical diagnoses among the patients were mostly cutaneous abscesses (n=41); folliculitis/furuncle/impetigo (n=35) and secondary infections of underlying dermatological conditions (n=35). The information regarding history of animal contact was available for only 25 participants, and all of them reported that they had no animal contact. Sixty-six patients were treated with antibiotics with a majority (n/N=36/66) receiving beta lactam drugs mainly amoxicillin-clavulanic acid (n=26), cefuroxime (n=6) or ceftriaxone (n=4). Fifteen patients received topical fusidic acid. **Table 1** shows the details of the demographic and clinical profiles of the patients.

**Table 1:** Demographic and Clinical Profile of patients with skin and soft tissue infections.

Total number of patients			N = 135
Age (mean ± SD)			27.7 ( ± 17.9) years
			n (%)
Gender	Male		64 (47.4%)
	Female		71 (52.6%)
Clinical diagnosis			
	Cutaneous/subcutaneous abscess		41 (30.4%)
	Folliculitis/furuncle/impetigo		35 (25.9%)
	Secondary infection of underlying dermatological condition		35 (25.9%)
	Cellulitis		14 (10.4%)
	Wound infection		10 (7.4%)
Recent antibiotic use			24 (17.8%)
	Antibiotics	*n*	
	Beta lactams	*10*	
	Fusidic acid	*6*	
	Fluoroquinolones	*2*	
	Unknown	*2*	
	Vancomycin, Mupirocin, Tetracycline, Erythromycin	*n= 1 each*	
Recent travel history			6 (4.4%)
Recent inset bite			4 (2.9%)
Treatment with antibiotics			66 (48.9%)
	*Antibiotics*	*n*	
	Beta lactams	*36*	
	Fusidic acid	15*	
	Macrolides	*10*	
	Mupirocin	5**	
	Fluroquinolones	*4*	
	Tetracyclines	*4*	
*Staphylococcus aureus*	Methicillin susceptible *S. aureus* (MSSA)		86 (66.7%)
identified (N=129)	Methicillin resistant *S. aureus* (MRSA)		43 (33.3%)
Outcome	Recovered		135 (100%)

*6 patients received fusidic acid in combination with either a beta lactam, macrolide or tetracycline **2 patients received mupirocin in combination with a macrolide.

### 3.1 Lateral Flow Immunoassay

After exclusion of negative cultures and duplicate strains, a total of 129 isolates (MRSA: n=43; MSSA: n=86) were tested by lateral flow immunoassay. Of these, 76 (58.9%) were unambiguously negative for PVL and these were the same isolates which were also negative for *pvl* genes as determined by DNA microarray. All 53 isolates which were positive using the lateral flow assay also had *pvl* genes detectable by DNA microarray. Using the molecular detection as gold standard, this translates into 100% each for diagnostic sensitivity, specificity and positive and negative predictive values of the lateral flow immunoassay. There were four isolates which yielded weak positive signals on the lateral flow immunoassay. Assuming that these could be misread as negative by unexperienced or distracted users in a real-life setting, a diagnostic sensitivity of 92.5%, a negative predictive value of 95% as well as specificity and positive predictive value of 100% can be expected. The test typically takes about 15 min inclusive of 10 min incubation period.

### 3.2 Molecular Characterization of *S. aureus* Isolates

All 129 isolates were successfully characterized and assigned to clonal complexes and strains. There was a wide clonal diversity with isolates being assigned to 28 clonal complexes (CC) and 58 strains (**Table 2**). The predominant CC were CC30 (n=18), CC22 (n=13), CC5 (n=12), CC1 (n=11), CC152 (n=8), CC15 (n=7); CC97 (n=7) as well as CC8 and CC20 (n=6 each) (**Table 2**). One isolate was determined to be *Staphylococcus argenteus* CC2696. The MRSA isolates were predominantly CC22 (n=11), CC30 (n=12) and CC5 (=8).

**Table 2:** Distribution of clonal complexes and strain assignment.

Total number of isolates characterized by DNA Microarray: 129			
Clonal complex	Number of isolates (%)	Strain Assignment	Number of isolates (%)
CC1	11 (8.5%)	CC1-MSSA	3 (2.3%)
		CC1-MSSA (PVL+)	1 (0.8%)
		CC1-MSSA-[fusC+ccrAB1]	5 (3.9%)
		CC1-MSSA-[fusC+ccrAB1] (PVL+)	1 (0.8%)
		CC1-MRSA-[IV+fusC+ccrAB1], WA MRSA-1/45	1 (0.8%)
CC5	12 (9.3%)	CC5-MSSA	2 (1.6%)
		CC5-MSSA (PVL+)	1 (0.8%)
		CC5-MSSA-[fusC+ccrC]	1 (0.8%)
		CC5-MRSA-[V/VT+fusC] (PVL+)	3 (2.3%)
		CC5-MRSA-[V/VT+fusC] (sed/j/r-)	4 (3.1%)
		CC5-MRSA-IV (PVL+/edinA+), WA MRSA-003/-121	1 (0.8%)
CC6	3 (2.3%)	CC6-MSSA	1 (0.8%)
		CC6-MSSA-[fusC+ccrC]	1 (0.8%)
		CC6-MRSA-IV, WA MRSA-51	1 (0.8%)
CC7	4 (3.1%)	CC7-MSSA	4 (3.1%)
CC8	6 (4.7%)	CC8-MSSA	3 (2.3%)
		CC8-MSSA-[kdp+bap]	1 (0.8%)
		CC8-MRSA-[IV+ACME] (PVL+), USA300	2 (1.6%)
CC10	1 (0.8%)	CC10-MSSA	1 (0.8%)
CC15	6 (4.7%)	CC15-MSSA	4 (3.1%)
		CC15-MSSA (PVL+)	1 (0.8%)
		CC15-MRSA-[V/VT+fusC]	1 (0.8%)
CC15 (ST199)	1 (0.8%)	CC15 (ST199)-MSSA	1 (0.8%)
CC20	6 (4.7%)	CC20-MSSA	6 (4.7%)
CC22	13 (10.1%)	CC22-MSSA	2 (1.6%)
		CC22-MRSA-IV (PVL+)	4 (3.1%)
		CC22-MRSA-IV (PVL+/tst+)	7 (5.4%)
CC25	1 (0.8%	CC25-MSSA	1 (0.8%)
CC30	18 (14.0%)	CC30-MSSA	6 (4.7%)
		CC30-MSSA (PVL+)	3 (2.3%)
		CC30-MRSA-IV (PVL+), “WSPP/Southwest Pacific Clone”	7 (5.4%)
		CC30-MRSA-[VI+fusC] (PVL+)	2 (1.6%)
CC45 [*agr* I]	2 (1.6%)	CC45-MSSA	2 (1.6%)
CC80	1 (0.8%)	CC80-MRSA-IV (PVL+)	1 (0.8%)
CC97	7 (5.4%)	CC97-MSSA	6 (4.7%)
		CC97-MRSA-[V/VT+fusC]	1 (0.8%)
CC101	1 (0.8%)	CC101-MSSA	1 (0.8%)
CC121	5 (3.9%)	CC121-MSSA	4 (3.1%)
		CC121-MRSA-V/VT (PVL+)	1 (0.8%)
CC152	8 (6.2%)	CC152-MSSA (PVL+)	7 (5.4%)
		CC152-MRSA-IV (PVL+)	1 (0.8%)
CC182	1 (0.8%)	CC182-MSSA	1 (0.8%)
CC188	2 (1.6%)	CC188-MSSA (PVL+)	2 (1.6%)
CC361	1 (0.8%)	CC361-MSSA	1 (0.8%)
CC398	2 (1.6%)	CC398-MSSA	1 (0.8%)
		CC398-MRSA-V/VT (PVL+)	1 (0.8%)
CC398 (ST291/813)	2 (1.6%)	ST291/813-MSSA	1 (0.8%)
		ST291/813-MSSA (PVL+)	1 (0.8%)
CC772	4 (3.1%)	CC772-MSSA (PVL+)	1 (0.8%)
		CC772-MRSA-V/VT (PVL+), “Bengal Bay Clone”	3 (2.3%)
CC1153	3 (2.3%)	CC1153-MSSA	1 (0.8%)
		CC1153-MRSA-PseudoSCCmec [class B+fusC+ccrAB1] (PVL+)	1 (0.8%)
		CC1153-MRSA-[V/VT+fusC] (PVL+)	1 (0.8%)
ST1156	1 (0.8%)	ST1156-MSSA	1 (0.8%)
CC2531	1 (0.8%)	ST2816-MSSA	1 (0.8%)
ST2867	3 (2.3%)	ST2867-MSSA	3 (2.3%)
CC2990	2 (1.6%)	CC2990-MSSA	2 (1.6%)
*Staphylococcus argenteus* CC2596	1 (0.8%)	CC2596-MSSA	1 (0.8%)

MSSA, Methicillin sensitive Staphylococcus aureus; MRSA, Methicillin resistant Staphylococcus aureus.

**Table 3** shows the distribution of virulence and antibiotic resistance genes in the isolates characterized. Commonly identified virulence genes were the *pvl* genes *lukS-PV* and *lukF-PV*, toxic shock syndrome toxin 1 (*tst1*) and various enterotoxin genes, *S. aureus* surface protein G (*sasG*) and epidermal cell differentiation inhibitor B (*edinB*) genes. Most of the MRSA strains (35/43; 81%) harbored the *lukS-PV* and *lukF-PV* genes. This was a much higher proportion compared to MSSA, out of which 18 isolates (21%) were positive for *lukS-PV* and *lukF-PV* genes. The fusidic acid resistance gene *fusC* was detected in 14 MRSA (33%) strains while only 8 MSSA (9%) were positive for this gene, 7 MRSA (CC5, CC30, CC1153) carried both, *fusC* and *pvl* genes. This combination was seen only once among MSSA (CC1). The mupirocin resistance (*mupA*) gene were detected in four strains (MSSA/MRSA: n=2 each) and none of the isolates was found to carry vancomycin resistance genes.

**Table 3:** Distribution of virulence and antibiotic resistance gene markers.

Selection of gene markers/cluster		# positive (N = 129)	% positive
Antibiotic resistance genes/locus			
Alternate penicillin binding protein 2, defining MRSA	*mecA*	43	33.3
Mercury resistance operon	*merA; merB*	1	0.8
Cassette chromosome recombinase A/B, type 1	*ccrA/B-01*	8	6.2
Cassette chromosome recombinase A/B, type 2	*ccrA/B-02*	25	19.4
Cassette chromosome recombinase A/B, type 3	*ccrA/B-03*	0	0.0
Cassette chromosome recombinase AA/C	*ccrAA/C*	17	13.2
Cassette chromosome recombinase A/B, type 4	*ccrA/B-04*	2	1.6
SCC*mec* XI	*mecC; blaZ-SCCmec XI*	0	0.0
Truncated methicillin resistance operon repressor 1	*delta_mecR1*	28	21.7
Beta-lactamase operon	*blaZ; blaI; blaR*	106	82.2
rRNA adenine N-6-methyl-transferase, erythromycin/clindamycin resistance	*ermA*	5	3.9
Erythromycin/clindamycin resistance	*ermB*	0	0.0
Erythromycin/clindamycin resistance	*ermC*	16	12.4
Lincosamide Nucleotidyltransferase	*linA*	4	3.1
Energy-dependent efflux of erythromycin	*msrA*	16	12.4
Macrolide efflux protein A	*mefA*	0	0.0
Lysylphosphatidyl glycerol synthetase	*mph(C)*	13	10.1
Bifunctional enzyme gentamicin resistance	*aacA-aphD*	18	14.0
Amino-glycoside adenyl-transferase,tobramycin resistance	*aadD*	10	7.8
3’5’-aminog-lycoside phospho-transferase, neo-/kanamycin resistance	*aphA3*	13	10.1
Streptothricine acetyltransferase	*sat*	13	10.1
Dihydrofolate reductase type 1	*dfrA*	10	7.8
Hypothetical protein associated with fusidic acid resistance	*Q6GD50 (fusC)*	22	17.1
Mupirocin resistance protein	*mupA*	4	3.1
Tetracycline resistance	*tetK*	8	6.2
Tetracycline resistance	*tetM*	2	1.6
Chloramphenicol acetyltransferase	*cat*	1	0.8
23S rRNA methyltransferase	*cfr*	1	0.8
Chloramphenicol/florfenicol exporter	*fexA*	1	0.8
Quaternary ammonium compound resistance protein A/B	*qacA*	1	0.8
Quaternary ammonium compound resistance protein A/B	*qacC*	2	1.6
Vancomycin resistance genes	*vanA; vanB*	0	0.0
Teicoplanin resistance gene from enterococci	*vanZ*	0	0.0
Virulence genes			
Toxic shock syndrome toxin 1	*tst1*	15	11.6
Panton Valentine leukocidin F/S component	*lukF-PV; lukS-PV*	53	41.1
Accessory gene regulator I	*agrI*	61	47.3
Accessory gene regulator II	*agrII*	32	24.8
Accessory gene regulator III	*agrIII*	30	23.3
Accessory gene regulator IV	*agrIV*	6	4.7
Capsule type 5	*cap5*	70	54.3
Capsule type 8	*cap8*	59	45.7
Exfoliative toxin serotype A	*etA*	2	1.6
Exfoliative toxin serotype B	*etB*	2	1.6
Enterotoxin A	*entA*	22	17.1
Enterotoxin A, allele from strain 320E	*entA (320E)*	0	0.0
Enterotoxin A, allele from strain N315 (Enterotoxin P)	*entA (N315)/entP*	8	6.2
Enterotoxin B	*seB*	15	11.6
Enterotoxin C	*seC*	14	10.9
Enterotoxin C	*seD*	1	0.8
Enterotoxin E	*seE*	0	0.0
Enterotoxin H	*seH*	13	10.1
Enterotoxin J	*seJ*	1	0.8
Enterotoxin K	*seK*	9	7.0
Enterotoxin L	*seL*	14	10.9
Enterotoxin Q	*seQ*	9	7.0
Enterotoxin R	*seR*	1	0.8
Enterotoxin gene cluster (total)	*egc*	64	49.6
Enterotoxin-like protein ORF CM14	*ORF CM14*	10	7.8
Exfoliative toxin D	*etD*	4	3.1
Epidermal cell differentiation inhibitor A	*edinA*	1	0.8
Epidermal cell differentiation inhibitor B	*edinB*	17	13.2
Epidermal cell differentiation inhibitor C	*edinC*	1	0.8
Arginine catabolic mobile element locus	*ACME*	2	1.6
*Staphylococcus aureus* surfaceprotein G	*sasG*	82	63.6

## 4 Discussion

We present the first report on the application of a novel lateral flow immunoassay for PVL detection in *S. aureus* isolates in a setting with wide MSSA/MRSA clonal diversity and high prevalence of *pvl* ^+ve^ strains. Using well-established DNA microarray-based assays for molecular detection of *pvl* genes as the gold standard, the findings show that the lateral flow immunoassay demonstrates excellent diagnostic accuracy indices. There was full concordance in *pvl* detection by the lateral flow immunoassay and the molecular confirmatory method used. As previously reported, the *in vitro* expression of PVL varies widely depending on CC affiliation of the *S. aureus* strain and the presence of other virulence genes (Stieber et al., 2014). This could explain the weak positive signals observed for four isolates. The 100% diagnostic sensitivity and specificity we have documented is of significance as this study was carried out in the context of a setting characterized by high prevalence of *pvl* ^+ve^ strains. Two similar assays have previously been reported that yielded similar high diagnostic accuracy indices using strains obtained from various countries (Badiou et al., 2010; Monecke et al., 2013). Unfortunately, these assays were eventually not marketed by the respective companies so the need for an easy, non-molecular culture confirmation test remained unaddressed. Our findings show that despite the remarkable evolution of *S. aureus* genotypes in recent years, the lateral flow immunoassay for PVL detection still yields excellent diagnostic results. This could be attributed to the rare occurrence of sequence variations in *pvl* genes. As these genes face a selective pressure to maintain their function in presence of the host´s defenses, a significant degree of conservation is to be expected (O’hara et al., 2008; Boakes et al., 2011). The lateral flow immunoassay is rapid, easy to use and interpret, and is highly economical making it ideal for diagnostic microbiology laboratories worldwide. It will be particularly beneficial in diagnostic laboratories serving primary care facilities where majority of patients with SSTIs are first seen (Ramakrishnan et al., 2015). Several countries in the developing world have reported a high prevalence of *pvl* ^+ve^ *S. aureus* strains and this lateral flow assay will be optimal in such resource limited settings where access to molecular assays may be lacking (Rasigade et al., 2010; Monecke et al., 2014; Abdulgader et al., 2015; Rasigade et al., 2015). With the potential threat of poor clinical outcomes associated with *pvl* ^+ve^ *S. aureus* strains, their early identification is crucial particularly in settings such as ours where *S. aureus* strains harboring *pvl* genes are circulating widely (Szmigielski et al., 1998; Kaneko and Kamio, 2004; Shallcross et al., 2013; Senok et al., 2020a). In this study, most of the patients had presentations consistent with mild SSTIs (Ramakrishnan et al., 2015) and majority received antibiotics with beta lactams and fusidic acid being the most prescribed antibiotics. According to current guidelines, mild purulent SSTIs in easily accessible sites with minimal cellulitis can be treated by incision and drainage alone, without a need for antibiotics (Public Health England, 2008; Gillet et al., 2011; Ramakrishnan et al., 2015). However, an antimicrobial coverage to mitigate against a potential threat of complications occurs commonly thus contributing to antibiotic misuse. The widespread utilization of beta lactams and fusidic acid in SSTI is a likely contributory factor to the emergence of resistance to these antibiotics resulting in the high occurrence of MRSA strains with SCC*mec* + SCC*fusC* composite elements in the Arabian Gulf region (Senok et al., 2020a; Monecke et al., 2021). The continued evolution and upward trend of MRSA in the region has been linked to strains harboring these composite elements (Senok et al., 2020a; Monecke et al., 2021). Hence, the high prevalence of MRSA isolates associated with SSTI, as well as the rate of detection of isolates harboring *pvl* and *fusC* genes is worrisome. The presence of co-carriage of *pvl* and *fusC* genes was found more commonly in MRSA compared to MSSA strains which is in keeping with reports of other studies from the same geographic region (Monecke et al., 2012b; Senok et al., 2016b; Boswihi et al., 2018; Senok et al., 2019b; Senok et al., 2020a). Therefore, it is envisaged that the incorporation of this lateral flow immunoassay in the diagnostic workflow will enable microbiology laboratories to provide clinicians with information on the PVL status of the MSSA/MRSA isolates identified from bacterial cultures of SSTIs which would be helpful in guiding physicians towards rational choices that support judicious use of antibiotics.

It is noteworthy that in contrast to other studies, the findings from this study provide a unique insight into the molecular epidemiology of *S. aureus* strains causing SSTIs in our setting. These strains harbor a diversity of antibiotic resistance genes including *mecA*, *fusC, aacA-aphD* and in addition to *pvl* they also carry important toxin virulence genes notably *tst-1*, *edinB, etA* and various enterotoxin genes. In the UAE and countries of the Arabian Gulf region, MRSA contributes significantly to the burden of clinical infections and most of the MRSA strains detected in this study are those that have previously been observed in the region (Senok et al., 2016a; Boswihi et al., 2018; Senok et al., 2020a). Although wide clonal and strain diversity was evident with 19 different MRSA strains belonging to 14 different clonal complexes identified, unlike other reports no novel variants were found in this study. Some of the strains identified have also been detected in other parts of the world which suggests the role of a dynamic population movement in facilitating either importation into the UAE, or dissemination to other countries by returnees.

Our findings demonstrate the contribution of well-known pandemic and regionally restricted as well as rare MRSA strains to these infections. These strains include CC1-MRSA-[IV+*fusC*+*ccrAB1*] which has previously been described in Western Australia as well as in the UAE and Saudi Arabia (Coombs et al., 2006; Raji et al., 2016; Senok et al., 2020a). It has also been detected in Pakistan (unpublished observation/personal communication, Dr B. Jamil, BJ Micro Lab (SMC Private) Limited, Pakistan and Dr. A. Syed, University of Haripur, Pakistan), a country with dynamic population movement to Arabian Gulf countries. The CC5 strains include CC5-MRSA-IV (PVL+/*edinA*+) which has been reported in the Arabian Gulf as well as in Australia, where it is, dubbed WA MRSA-121 (Riley and Rouse, 1995; Dailey et al., 2005; Bloomfield et al., 2020). The CC5-MRSA-[V/VT+*fusC*] was found four times; one isolate was subtyped and yielded SCC*mec* V rather than VT. This strain which appears to be common in this region was sporadically found in Australia (WA MRSA-109) (Coombs et al., 2012; Senok et al., 2020a). The CC5-MRSA-[V/VT+*fusC*] (PVL+) has been identified sporadically at the Gulf and in Australia (Senok et al., 2020a). The CC6-MRSA-IV is common in the Middle East and has also been found in Europe among refugees of Middle Eastern origin (Monecke et al., 2011a; Monecke et al., 2012b; Kossow et al., 2018; Bartels et al., 2021) as well as in Australia.

CC8-MRSA-[IV+ACME] (PVL+), USA300 is a well-known community-acquired pandemic MRSA strain which is abundant in North America and sporadic in Western Europe but was recently observed in Pakistan and Afghanistan (Monecke et al., 2011a). In the UAE, novel variants of this MRSA strain and putative PVL-deletion mutants have been reported (Senok et al., 2020a). For CC15-MRSA-[V+*fusC*] the ancestral CC15-MSSA is a common nasal colonizer in the Middle East (Sarkar et al., 2016; Senok et al., 2018). However, in recent years, CC15-MRSA has emerged among humans and livestock (camels) in the UAE, Kuwait and Saudi Arabia (Raji et al., 2016; Senok et al., 2017; Boswihi et al., 2018; Senok et al., 2020a). CC22-MRSA are prevalent in the Arabian Gulf region and six distinct ‘strains’ of CC22-MRSA-IV have been reported (Senok et al., 2016b). Whilst CC22-MRSA-IV (PVL+) are common in the Middle East, they occur sporadically in other regions. CC22-MRSA-IV (PVL+/*tst+*) was apparently first observed in Iran (Goudarzi et al., 2017) and spread to other Gulf countries as well as far as to Nepal (Roberts et al., 2018; Roberts et al., 2020). The recent identification of CC22-MRSA-[IV + *fusC* + *ccrAA/(C*) which harbored a novel SCC*mec* element is indicative of the expansion of the diversity of CC22-MRSA in the region (Senok et al., 2020a). CC30-MRSA-IV (PVL+) also known as “WSPP/Southwest Pacific Clone” was first described from Australia and Pacific islands (Monecke et al., 2011a). However, there are different variants with different SCC*mec* subtypes, and this might indicate a polyphyletic origin, i.e., multiple acquisitions of SCC*mec* elements by ancestral CC30 MSSA. This is not fully understood and might warrant further study. However, the CC30-MRSA-[VI+*fusC*] (PVL+) detected in this study appears to be uncommon and restricted to the Arabian Gulf region (Senok et al., 2019a).

CC80-MRSA-IV (PVL+) was frequently dubbed the “European Clone” of community-acquired MRSA but recently it emerged in the Maghreb countries (Algeria, Tunisia) and Lebanon from where the possibility of dissemination to Arabian Gulf countries is feasible due frequent travel (Kechrid et al., 2011; Djoudi et al., 2013; Basset et al., 2015).

CC97-MRSA-[V +*fusC*] is a Middle Eastern strain observed in Saudi Arabia and the UAE (Senok et al., 2016a; Senok et al., 2020a). However, sporadic cases from Western Europe have been reported as well as an outbreak in Ireland which was linked to an Egyptian connection (Mcmanus et al., 2021). CC121-MRSA-V/VT (PVL+) are rare in the UAE (Senok et al., 2020a) and elsewhere. Another rare strain is CC152-MRSA-IV (PVL+) which had previously been observed in Kuwait (Boswihi et al., 2018). CC398-MRSA-V/VT (PVL+) is related to the European livestock strain. However, the PVL-positive lineage has no livestock connection, and it is known to occur in South-East Asia (Yu et al., 2008; Moller et al., 2019). Indeed, cases of infections and outbreaks associated with this CC398-MRSA in Europe were linked to South-East Asia (Moller et al., 2019; Nurjadi et al., 2019). The first identification of this strain in the Middle East region was recently reported in the UAE, possibly introduced *via* South-East Asia or Europe (Senok et al., 2020a). CC772-MRSA-V (PVL+), “Bengal Bay Clone” is a pandemic, multi-resistant and virulent strain emerging from the Indian subcontinent (i.e., India, Bangladesh, Pakistan) (Ellington et al., 2010; Steinig et al., 2019). Given the high number of people from these countries working in the region, its occurrence is not surprising, and indeed, it has been observed in many studies from the region (Monecke et al., 2012a; Udo et al., 2014; Boswihi et al., 2016; Senok et al., 2020b). CC1153-MRSA-[V/VT+*fusC*] (PVL+) and CC1153-MRSA-PseudoSCC*mec* [class B+*fusC*+*ccrAB1*] (PVL+) are both strains that are known to occur mainly in Gulf countries (Monecke et al., 2021).

With regards to MSSA, a wide variety of different strains was found including some rare and poorly characterized lineages such as CC1156, CC2531, CC2867, CC2990 and *S. argenteus* CC2596. The most common *pvl* ^+ve^ MSSA strain was CC152-MSSA (7 isolates), a lineage previously known to occur in Western Africa and Trinidad & Tobago (Ruimy et al., 2008; Monecke et al., 2014; Okuda et al., 2016; Obasuyi et al., 2020).

In conclusion, we demonstrate excellent diagnostic accuracy indices for a novel lateral flow immunoassay for rapid identification of PVL in MSSA/MRSA identified from cultures of SSTI in a setting with high prevalence of *pvl* ^+ve^ strains. These findings support the introduction of this test for routine use in the diagnostic microbiology laboratory especially in settings where facilities for molecular assays are lacking. The prevalence and diversity of MRSA strains associated with SSTIs as well as the high occurrence of *pvl* and *fusC* genes in these strains is of concern.

## Data Availability Statement

The raw data supporting the conclusions of this article will be made available by the authors, without undue reservation.

## Ethics Statement

The studies involving human participants were reviewed and approved by Mohammed Bin Rashid University and Mediclinic Hospitals ethics committees (MBRU-IRB-2020-003 and MCME.CR.98.MCIT.2020). Written informed consent for participation was not required for this study in accordance with the national legislation and the institutional requirements.

## Author Contributions

Conceptualization, AS, SM, and RE. Data curation, AS, SM, RN, HC, ST, EM, and RE. Formal analysis, AS, SM, and RE. Investigation, RN, HC, ST, and EM. Methodology, AS, SM, RN, HC, ST, EM, and RE. Project administration, AS, SM, and RN. Resources, AS, SM, and RE. Writing – original draft, AS, SM, and RE. All authors contributed to the article and approved the submitted version.

## Funding

We acknowledge the German Federal Ministry for Economic Affairs and Energy for supporting the development of the PVL antibodies and of the rapid assay within the framework of the INNO-KOM project (49MF180153).

## Conflict of Interest

The authors declare that the research was conducted in the absence of any commercial or financial relationships that could be construed as a potential conflict of interest.

## Publisher’s Note

All claims expressed in this article are solely those of the authors and do not necessarily represent those of their affiliated organizations, or those of the publisher, the editors and the reviewers. Any product that may be evaluated in this article, or claim that may be made by its manufacturer, is not guaranteed or endorsed by the publisher.
